# Differential microRNAs expression profiles in liver from three different lifestyle modification mice models

**DOI:** 10.1186/s12864-021-07507-3

**Published:** 2021-03-19

**Authors:** Huan Gong, Ming Zhang, Yiwen Han, Ying Zhang, Jing Pang, Yanyang Zhao, Beidong Chen, Wei Wu, Ruomei Qi, Tiemei Zhang

**Affiliations:** 1 The Key Laboratory of Geriatrics, Beijing Institute of Geriatrics, Beijing Hospital, National Center of Gerontology, National Health Commission; Institute of Geriatric Medicine, Chinese Academy of Medical Sciences, Beijing, 100730 People’s Republic of China; 2 Chinese People’s Liberation Army General Hospital, Beijing, People’s Republic of China; 3 School of Sport Science, Beijing Sport University, Beijing, People’s Republic of China

**Keywords:** Caloric restriction, Exercise, High-fat diet, microRNA, Lifestyle modifications

## Abstract

**Background:**

MicroRNAs play an important role in many fundamental biological and pathological processes. Defining the microRNAs profile underlying the processes by beneficial and detrimental lifestyles, including caloric restriction (CR), exercise and high-fat diet (HF), is necessary for understanding both normal physiology and the pathogenesis of metabolic disease. We used the microarray to detect microRNAs expression in livers from CR, EX and HF mice models. After predicted potential target genes of differentially expressed microRNAs with four algorithms, we applied GO and KEGG to analyze the function of predicted microRNA targets.

**Results:**

We describe the overall microRNAs expression pattern, and identified 84 differentially expressed microRNAs changed by one or two or even all the three lifestyle modifications. The common and different enriched categories of gene function and main biochemical and signal transduction pathways were presented.

**Conclusions:**

We provided for the first time a comprehensive and thorough comparison of microRNAs expression profiles in liver among these lifestyle modifications. With this knowledge, our findings provide us with an overall vision of microRNAs in the molecular impact of lifestyle on health as well as useful clues for future and thorough research of the role of microRNAs.

**Supplementary Information:**

The online version contains supplementary material available at 10.1186/s12864-021-07507-3.

## Background

Overweight and obesity have been recognized as risk factors for many chronic diseases such as the metabolic syndrome, diabetes, and cardiovascular diseases [1]. The main driver for weight gain is considered to be the medium or long term positive energy balance, usually through consumption of a high-fat diet (HF) [2, 3]. Treatment of obesity therefore often consists of reducing caloric intake or promoting energy utilization to diminish the surplus of energy in the system [3]. In contrast to the detrimental effects of overeating energy-dense foods, caloric restriction (CR), restricting the intake of calories without causing malnutrition, has a wide range of benefits, including promoting lifespan, decreasing the incidence of age-related diseases and extending health span as well [4]. On the other hand, physical activity and exercise are key approaches of energy expenditure and therefore of energy balance [5]. Exercise (EX) also confers multiple beneficial effects on health, such as the prevention of several cardiac and metabolic diseases [6]. CR, EX and HF converge on some common pathways, such as insulin signaling pathways and sirt1. Their contributions are also profoundly heterogeneous. The underlying common or unique mechanisms of CR, EX and HF have not yet been well understood. Identification of factors involved in them brings a promise of translatability to human health.

Genes (mRNA) involved in the process and intervention of obesity have been studied. However, the role of finer post-transcriptional gene regulatory mechanisms has not been comprehensively explored. MicroRNAs are a class of short non-coding RNAs which primarily interact with 3′ untranslated region (3’UTR) of mRNA, leading to either translational repression or mRNA degradation [7]. These small molecules regulate approximate one third of the protein-coding genes, therefore directly or indirectly involve in almost all cellular pathways [8]. The numerous roles of microRNAs have been demonstrated in many life processes such as metabolism, exercise and in general, physiological and pathological states [9–11]. The liver is a fundamental organ for diverse physiological processes, such as macronutrient metabolism, glucose, lipid and cholesterol homeostasis. Liver provides the energy needed to drive the aforementioned processes by processing, partitioning, and metabolism of macronutrients [12]. Defining the microRNAs profile underlying the control of hepatic functions and processes by CR, EX and HF is necessary for understanding both normal physiology and the pathogenesis of metabolic disease.

Recent years, the number of microRNA profiling studies has increased rapidly. MicroRNAs profiles in several different tissues were investigated after CR, EX or HF, including adipose tissue, skeletal muscle, heart, especially circulating microRNAs [13–16]. There are only few microRNAs profiling studies in liver under these lifestyle modification conditions. The aim of this study was to compare the effects of these conditions on microRNAs and identify the predominant microRNAs in mouse liver involved in these lifestyles. We performed microRNA analysis by microarray and validated the microRNA candidates by reverse transcription quantitative real-time polymerase chain reaction (RT-qPCR). To elucidate post-transcriptional regulation by these microRNAs, we analyzed the in silico predicted targets of the microRNAs by pathway enrichment analysis. Subsequently, we performed RT-qPCR analysis of selected targets.

## Results

### Establishment of lifestyle modification mice models

**Fig. 1:**
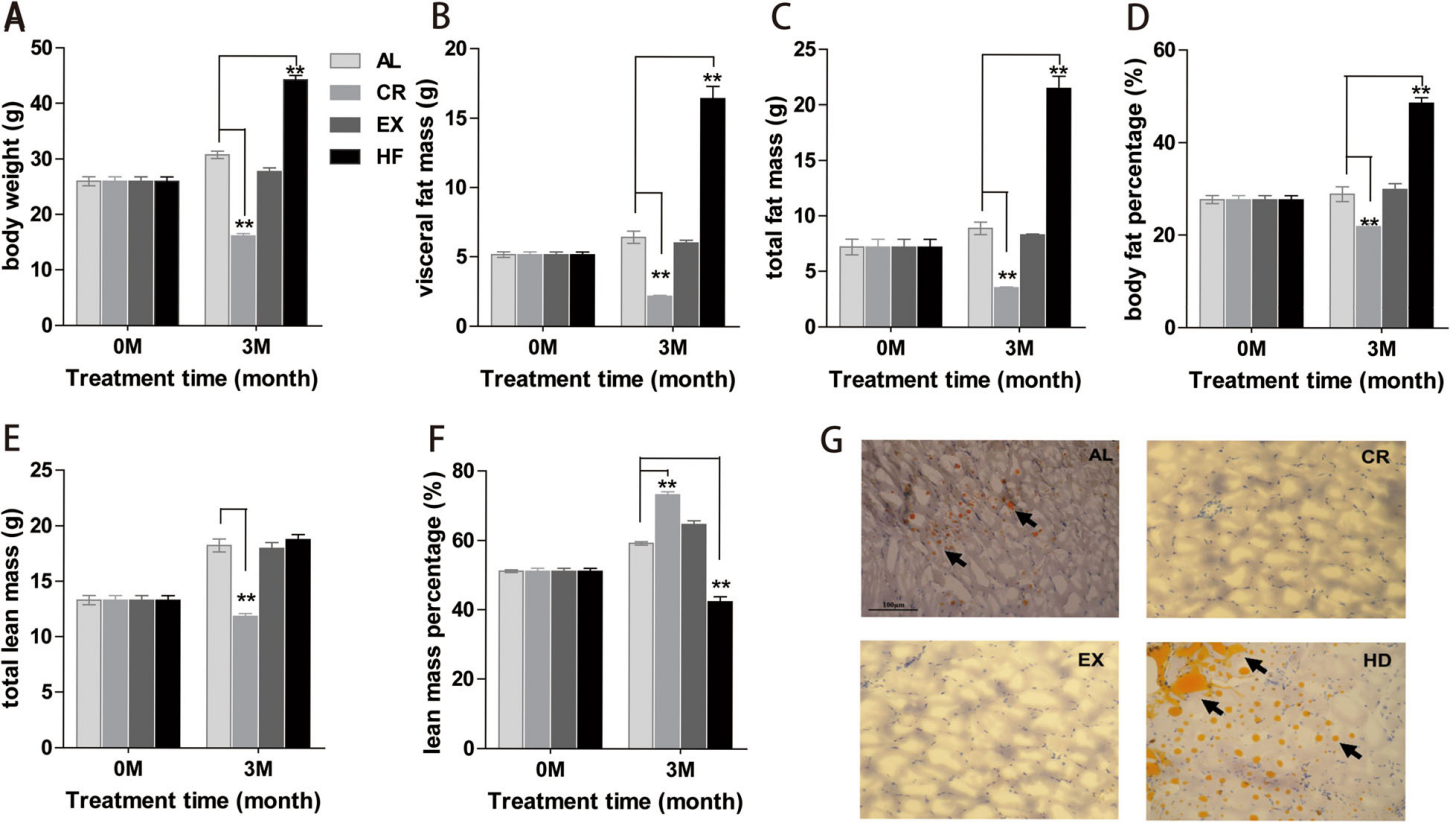
The assessment of lifestyle modification mice model establishment. Body weight of different lifestyle groups at the beginning and the end of the treatment (**a**). Visceral fat mass (**b**), total fat mass (**c**) and total lean mass (**e**) were detected by DEXA at the beginning and the end of the treatment. Body fat percentage (**d**) and lean mass percentage (**f)** were calculated. The frozen-fixed mice skeletal muscle was cut into 20 μm sections and stained by Oil Red O (**g**). Black arrows indicated the ectopic lipid accumulations. Data are presented as Means ± SE (n = 7 in each group). AL: ad libitum, CR: caloric restriction, EX: exercise, HF: high-fat diet. (**: p < 0.01 vs AL)

After treatment for 3 months, the body weight (16.2 ± 1.05 g), visceral fat mass (2.18 ± 0.15 g), total fat mass (3.55 ± 0.17 g) and total lean mass (11.86 ± 0.67 g) in CR group are significantly lower than in AL group (30.8 ± 1.77 g, 6.43 ± 1.16 g, 8.91 ± 1.50 g and 18.26 ± 1.54 g, respectively) (*p* < 0.01) (Fig. 1a-c, e). On the opposite, in HF group, the body weight (44.3 ± 2.12 g), visceral fat mass (16.43 ± 2.31 g) and total fat mass (21.51 ± 2.93 g) are significantly heavier than in AL group (p < 0.01) (Fig. 1a-c, e); total lean mass (18.81 ± 1.2 g) has no significant difference with AL group. While in EX group, the body weight (27.8 ± 1.84 g), visceral fat mass (6.03 ± 0.50 g), total fat mass (8.33 ± 0.20 g) and lean mass (18.01 ± 1.39 g) all have no significant differences with AL group (Fig. 1a-c, e). Body fat percentage has similar pattern as body weight, visceral fat mass and total fat mass (Fig. 1d). However, body lean mass percentage in CR group (73.2 ± 2.4%) is higher than in AL group (59.3 ± 1.3%, *p* < 0.01), and in HF group it is lower (42.5 ± 3.7%, p < 0.01) than in AL group. In EX group (64.8 ± 2.7%), it also has no significant difference with AL group (Fig. 1f). Furthermore, there are obvious ectopic lipid accumulations in skeletal muscle after high-fat diet feeding, while the ectopic lipid accumulations decrease in CR and EX group compared with AL mice (Fig. 1g). These results indicated that these lifestyle modifications induced corresponding effects on mice and the models were established successfully.

### Comprehensive microRNA profiling in livers from lifestyle modification mice models

**Fig. 2:**
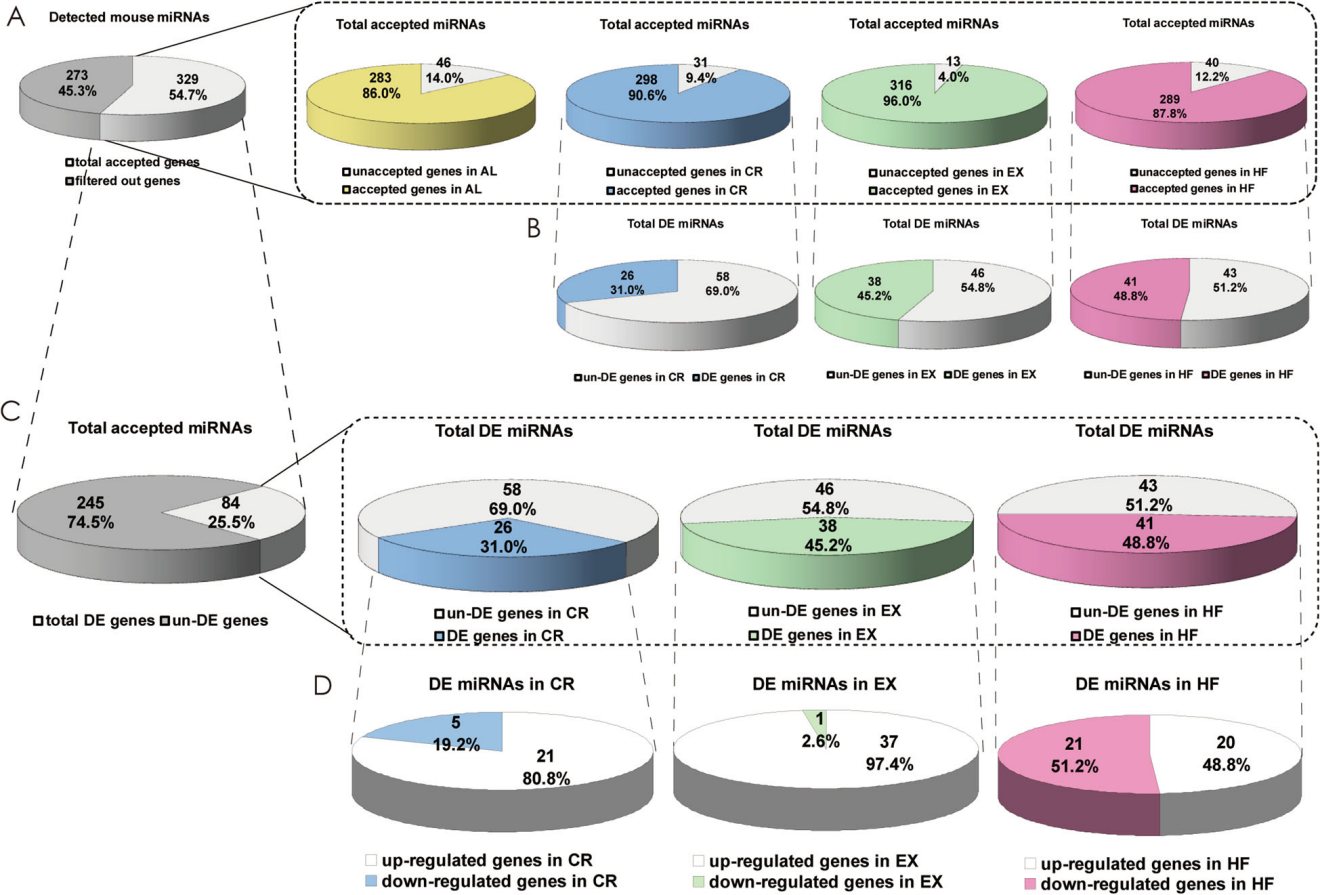
Numbers and ratios of detected (**a**), accepted (**a**, **b**) and differentially expressed (**c**, **d**) microRNAs. The profiling by microRNA array identified a subset of microRNAs that were differentially expressed. The intensity of green signal on the chip were calculated after background subtraction and replicated spots on the same slide have been averaged by getting a median intensity. Median Normalization Method was used to obtain “Normalized Data”, Normalized Data = (Foreground-Background)/median, the median is 50% quantile of microRNA intensity which is larger than 50 in all samples after background correction. The low intensity differentially expressed microRNAs were filtered (Foreground-Background intensities of which are all < 50 in two compared samples) then we got accepted microRNAs. The threshold value used to screen Up and Down regulated microRNAs is Fold Change> = 1.5 compared to AL group. AL: ad libitum, CR: caloric restriction, EX: exercise, HF: high-fat diet, DE: differentially expressed

To determine if microRNAs are involved in the process and function of lifestyle modification in liver, we analyzed differential expressed (DE) microRNAs using microarray technique. A total of 601 mature mouse microRNAs were profiled from the livers. Among them, 328 microRNAs were accepted as expressed genes in liver after filtering and were subjected to DE microRNAs analysis (Fig. 2a and Fig. S1, as described in Methods). There were least microRNAs accepted in AL and HF groups, 283 and 289 microRNAs, respectively; and most microRNAs accepted in EX group (316) (Fig. 2a). In all the accepted microRNAs in CR group, there were only 8.7% (26 microRNAs) differentially expressed compared to in AL group; there were larger proportion of DE microRNAs in EX (12.0%, 38microRNAs) and HF group (13.5%, 39microRNAs) than in CR group (Fig. 2b). Of all the 328 accepted microRNAs, there were only 25.6% (84 microRNAs) expressed differentially after lifestyle modifications in total (Fig. 2c): 31% (26 microRNAs) were from CR group, 45.2% (48 microRNAs) were from EX group and 46.4% (39 microRNAs) from HF group. Among DE microRNAs in CR group, 80.8% were found to be up-regulated and only 5 microRNAs were identified down-regulated; in EX group, only one microRNA was down-regulated; however, in HF group, there were almost equal up- and down-regulated microRNAs, 20 and 19 microRNAs respectively (Fig. 2d). These data suggested that microRNAs indeed involved in lifestyle modifications, however only a subset microRNAs function in liver and only a small portion of microRNAs involved in lifestyle modifications.

**Fig. 3:**
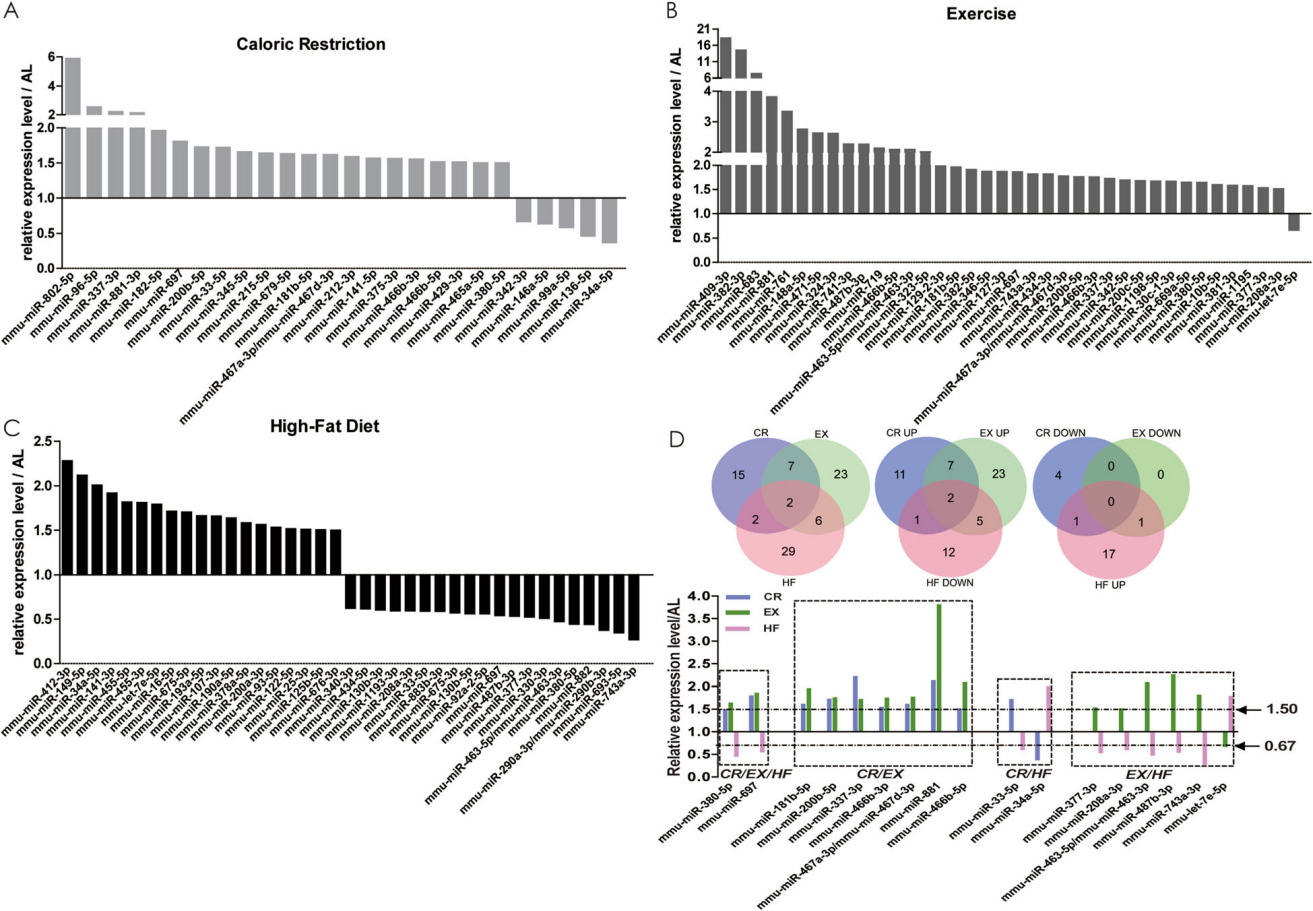
Differentially expressed microRNAs in different lifestyle modification mice models identified by microRNA array. After normalized and filtered as mentioned in Methods, DE microRNAs were identified. The threshold value used to screen Up and Down regulated microRNAs is Fold Change> = 1.5 compared to AL group. DE microRNAs in each lifestyle modification mice model were shown in **a, b** and **c.** The numbers and names of DE microRNAs existed in two or three lifestyle modification mice models were shown in **d**. AL: ad libitum, CR: caloric restriction, EX: exercise, HF: high-fat diet, DE: differentially expressed

The DE microRNAs in each group were shown in Fig. 3a-c and Fig. S1. Most of the DE microRNAs changed moderately. For the up-regulated microRNAs, only 4 out of 21, 14 out of 37 and 3 out of 20 genes were more than 2 folds in CR, EX and HF group, respectively. The range was only up to 2.28 folds in HF group; in CR group, only one microRNA was over 5 folds (5.90); the most changed microRNAs existed in EX group, in which there were 2 microRNAs were over 10 folds. On the other hand, for the down-regulated microRNAs, only 2 out of 5 and 6 out of 21 microRNAs were less than 0.5 folds in CR and HF group, respectively; the range was only as low as 0.37 and 0.27 folds in CR and HF group, respectively. Interestingly, there were several microRNAs altered by more than one lifestyle modifications (Fig. 3d): mmu-miR-380-5p and mmu-miR-697 were up-regulated by CR and EX and down-regulated by HF; seven microRNAs were up-regulated by both CR and EX; six microRNAs were oppositely altered by EX and HF and two by CR and HF. These results suggested that the changes of microRNAs after lifestyle modifications were fine-tuning in general and these lifestyle modifications impacted through both some common pathways and different pathways as well.

**Fig. 4:**
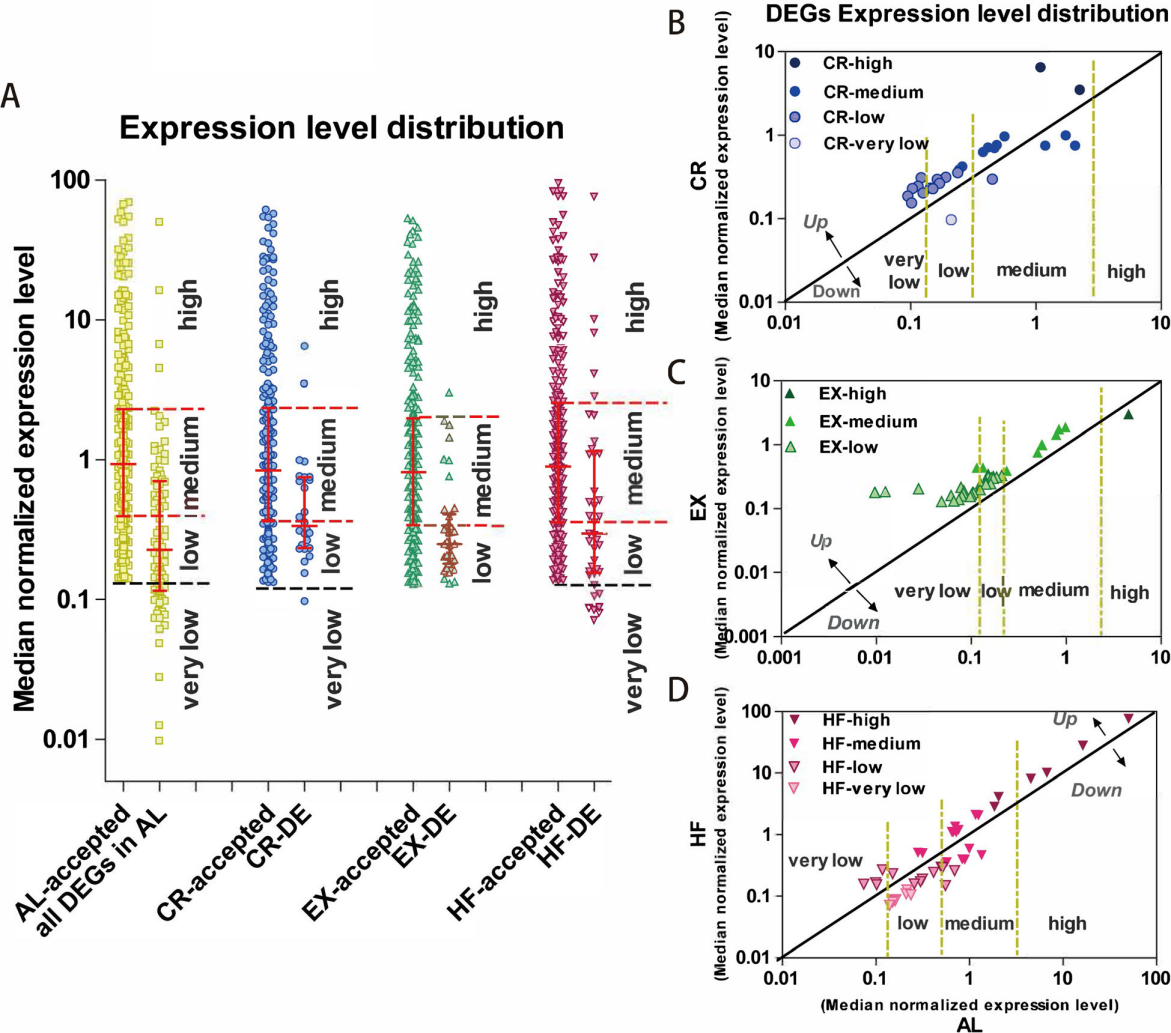
Expression level distributions of the accepted microRNAs and differentially expressed microRNAs. **a**. Median normalized expression levels of all the microRNAs after normalized and filtered in each group and the DE microRNAs in each group. “All DEGs in AL” shows the expression levels in AL group of the microRNAs differentially expressed in CR, EX or HF. In each group, the top 25% accepted microRNAs were taken as high abundant genes, the bottom 25% as low abundant genes and the middle 50% as medium abundant genes. Those with Foreground-Background intensities < 50 were taken as very low abundant genes. Line in scatter dot plot is at median with interquartile range. **b-d.** The DE microRNAs median normalized expression levels in the two compared groups. AL: ad libitum, CR: caloric restriction, EX: exercise, HF: high-fat diet, DEG: differentially expressed microRNA genes, Up: up-regulated microRNAs, Down: down-regulated microRNAs

After background correction and the very low intensity microRNAs filtration as described in Methods, in each group, the top 25% accepted microRNAs were taken as high abundant microRNAs, the bottom 25% as low abundant microRNAs and the middle 50% as medium abundant microRNAs. Those with Foreground-Background intensities < 50 were taken as very low abundant genes. In general, more than 50% DE microRNAs are low abundant genes in all the groups; only 4.8, 7.7, 2.6 and 15.4% DE microRNAs are high abundant genes in AL, CR, EX and HF group, respectively (Fig. 4a). 92.3% (24 of 26) DE microRNAs changed after CR have only medium to low or even very low abundance in both CR and AL groups, and almost half (12 of 26) DE microRNAs have low or very low abundance in both groups. Among them, 5 of 21 up-regulated microRNAs in CR have low abundance in CR and very low in AL group, and 1 of 5 down-regulated microRNAs after CR has low abundance in AL and very low in CR group (Fig. 4b). Among the DE microRNAs changed after EX, only 1 microRNA has high abundance and 5 had medium abundance in both EX and AL groups; almost half (18 of 37) up-regulated microRNAs after EX are low abundant genes in EX and very low in AL group (Fig. 4c). On the other hand, almost half (18 of 39) DE microRNAs in HF have medium to high abundance in both HF and AL groups; 4 of 19 up-regulated microRNAs by HF have low abundance in HF and very low in AL group; and 7 of 20 down-regulated microRNAs by HF have low abundance in AL and very low in HF group (Fig. 4d). The expression level distribution of DE microRNAs suggested that microRNAs with low and medium abundance were more susceptible to lifestyle modifications than those high abundant microRNAs.

### Validation of selected differentially expressed microRNAs via RT-qPCR

**Fig. 5:**
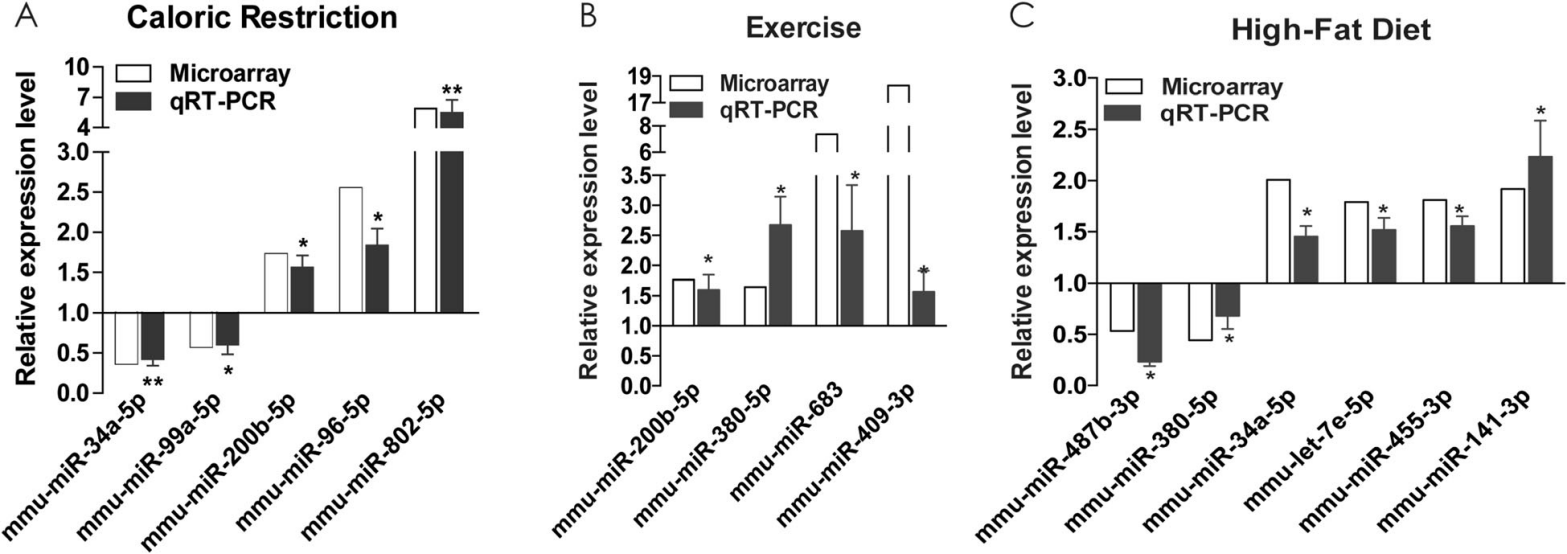
RT-qPCR validations of selected differentially expressed microRNAs in the livers of lifestyle modification mice models. The relative expression level of microRNAs in livers of CR (**a**), EX (**b**) and HF (**c**) were detected by PT-qPCR and normalized to U6, and the expression levels of AL mice were set at a relative expression of 1. RT-qPCR data were represented as mean ± SE and compared with microarray results. (*: *p* < 0.05, **: *p* < 0.01 and *** *p* < 0.001 vs AL. *n* = 5 in each group) AL: ad libitum, CR: caloric restriction, EX: exercise, HF: high-fat diet

Representative microRNAs were validated in an independent platform - RT-qPCR, including DE microRNAs in all the three lifestyle modifications, such as such as mmu-miR-34a-5p, mmu-miR-99a-5p, mmu-miR-200b-5p, mmu-miR-96-5p and mmu-miR-802-5p in CR group (Fig. 5a), mmu-miR-200b-5p, mmu-miR-380-5p, mmu-miR-683 and mmu-miR-409-3p in EX group (Fig. 5b), and mmu-miR-487b-3p, mmu-miR-380-5p, mmu-let-7e-5p, mmu-miR-455-3p and mmu-miR-141-3p in HF group (Fig. 5c). The RT-qPCR results showed similar direction of expression change as observed in microarray results.

### Functional prediction of differentially expressed microRNAs

To better understand the function of DE microRNAs in livers after lifestyle modifications, it is essential to identify their target genes. In this study, as described in Methods, we used four softwares to predict target genes and the intersections of the output results of at least three algorithms were used as prediction results for the DE microRNAs. These in silico predicted targets included mRNAs from liver and non-liver cell and tissue types. Therefore, to further identify tissue-specific target genes, the PaGenBase database was used to filter the predicted targets. A total of 853 mRNAs were identified as potential targets for the total 84 DE microRNAs from the three treatments.

**Fig. 6:**
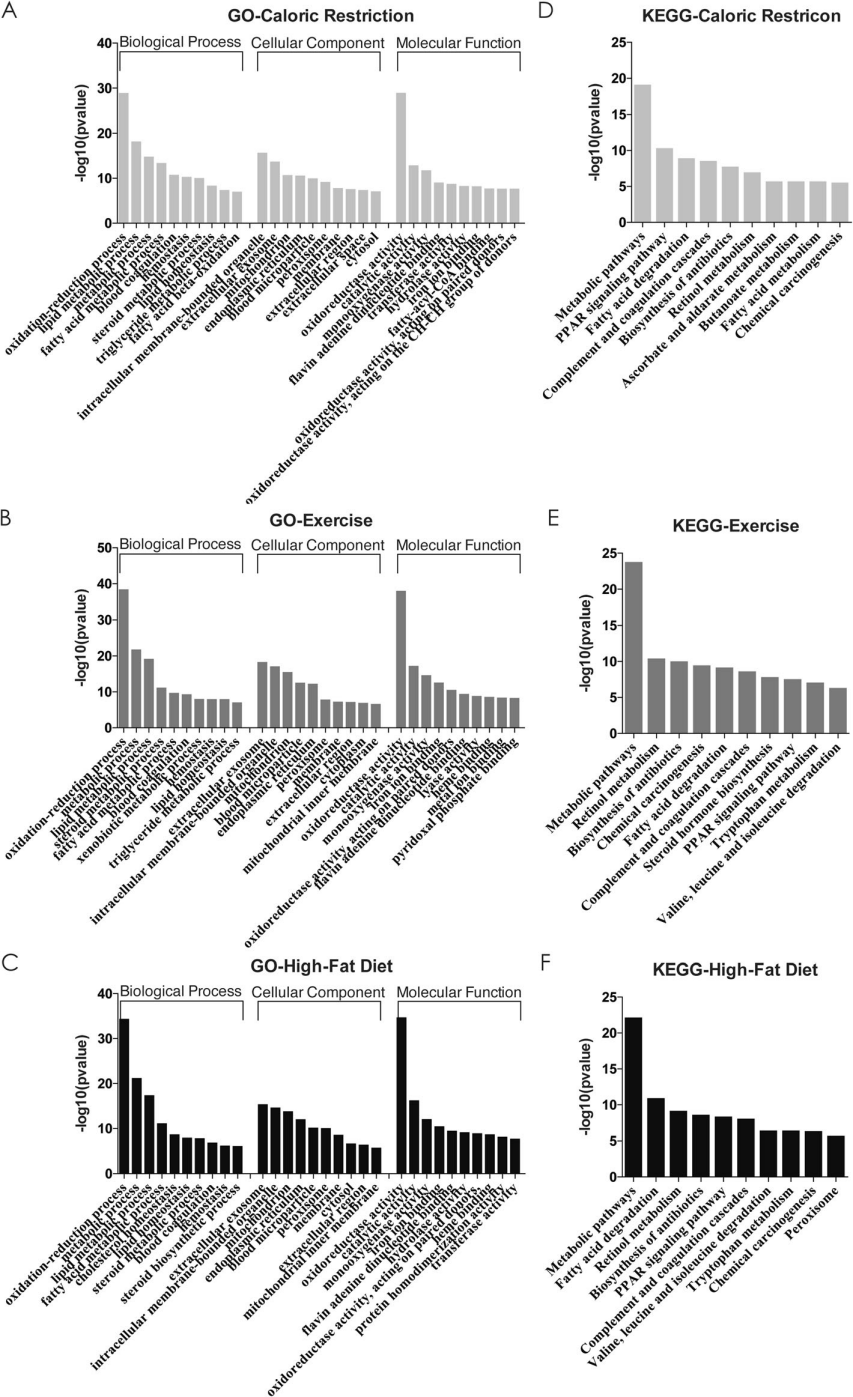
GO (A-C) and KEGG (D-F) analysis of target genes of differentially expressed microRNAs. The top 10 most enriched MFs, BPs and CCs GO term of predicted mRNA targets of differentially expressed microRNAs in livers of CR (**a**), EX (**b**) and HF (**c**). The top 10 most enriched KEGG pathways of predicted mRNA targets of differentially expressed microRNAs in livers of CR (**d**), EX (**e**) and HF (**f**). AL: ad libitum, CR: caloric restriction, EX: exercise, HF: high-fat diet

To determine the functions and connections of the DE microRNAs in these lifestyle modification mice models, we applied enrichment analyses to clarify the biological function of microRNA integrated-signature via target genes. Based on the distribution of the predicted target genes in the Gene Ontology analysis [17], the number of genes was statistically analyzed with significant enrichment of each GO term to elucidate gene function in biological process (BP), cellular component (CC) and molecular function (MF), and the results are shown in Fig. 6a-c. KEGG consists of databases with information about genomes, biological pathways, diseases, drugs, and chemical substances [18]. The top 10 pathways enriched by the candidate target genes are also displayed in histograms (Fig. 6d-f). In the top 10 enriched GO terms and KEGG pathways, the common and different enriched GO terms and KEGG pathways in these lifestyle modifications were listed in Table 1. These most striking categories of gene function and main biochemical and signal transduction pathways will point us in the direction of further research about DE microRNAs.

**Table 1:** The top 10 enriched GO terms and KEGG pathways

Group	GO terms			KEGG pathways
	BP	CC	MF	
Common in CR, EX and HF	oxidation-reduction process; lipid metabolic process; metabolic process; fatty acid metabolic process; blood coagulation; hemostasis; steroid metabolic process; lipid homeostasis	intracellular membrane-bounded organelle; extracellular exosome; mitochondrion; endoplasmic reticulum; blood microparticle; peroxisome; membrane; extracellular region	oxidoreductase activity; catalytic activity; monooxygenase activity; flavin adenine dinucleotide binding; iron ion binding; oxidoreductase activity (acting on paired donors, with incorporation or reduction of molecular oxygen)	Metabolic pathways; PPAR signaling pathway; Fatty acid degradation; Complement and coagulation cascades; Biosynthesis of antibiotics; Retinol metabolism; Chemical carcinogenesis
Common in CR and EX	triglyceride metabolic process	–	–	–
Common in CR and HF	–	cytosol	transferase activity; hydrolase activity	–
Common in EX and HF	–	mitochondrial inner membrane	heme binding	Tryptophan metabolism; Valine, leucine and isoleucine degradation
Only in CR	fatty acid beta-oxidation	extracellular space	fatty-acyl-CoA binding; oxidoreductase activity (acting on the CH-CH group of donors)	Ascorbate and aldarate metabolism; Butanoate metabolism; Fatty acid metabolism
Only in EX	xenobiotic metabolic process	cytoplasm	lyase activity; metal ion binding; pyridoxal phosphate binding	Steroid hormone biosynthesis
Only in HF	cholesterol homeostasis, steroid biosynthetic process	–	protein homodimerization activity	Peroxisome

### Validation of selected target mRNAs via RT-qPCR

**Fig. 7:**
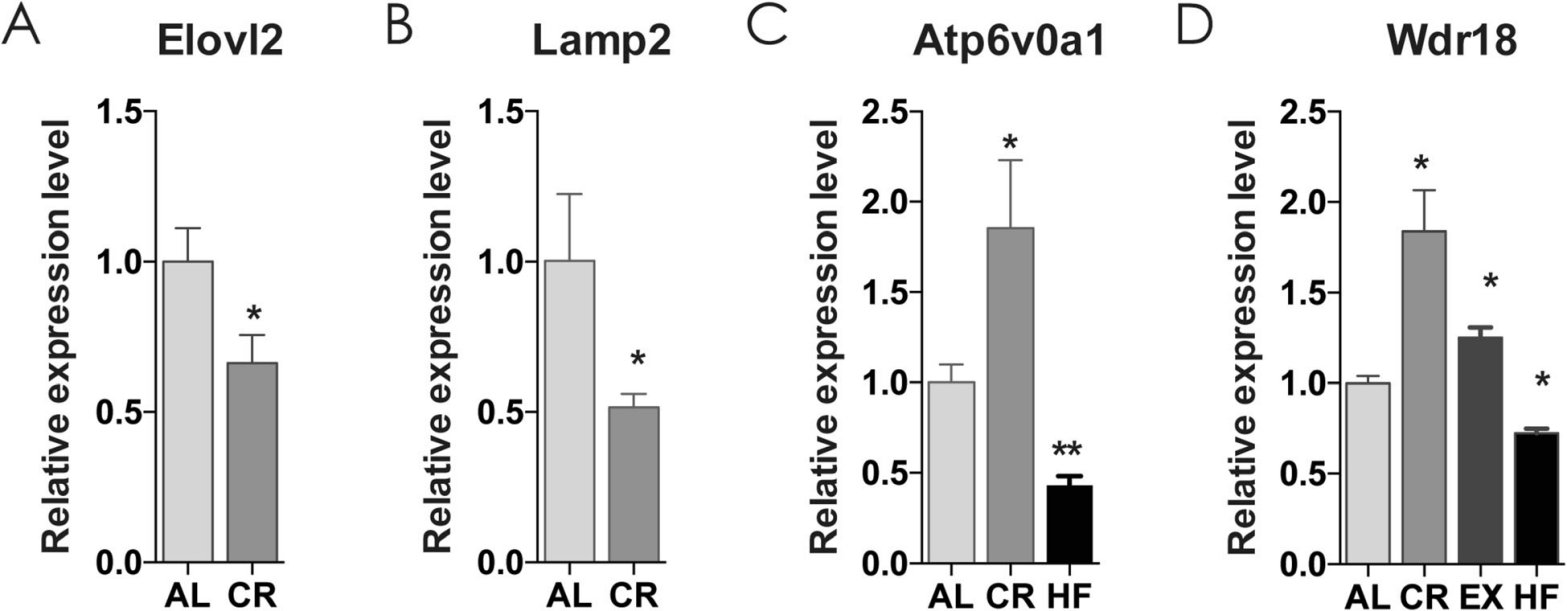
RT-qPCR validations of several predicted mRNA target of differentially expressed microRNAs. The relative expression level of Elovl2 (**a**), Lamp2(**b**), Atp6v0a1(**c**) and Wdr18(**d**) in liver indicated groups detected by PT-qPCR and normalized to Hmbs, and the expression levels of AL mice were set at a relative expression of 1. RT-qPCR data are represented as mean ± standard error (mean ± SE). (*: p < 0.05 and **: p < 0.01 vs AL. n = 5 in each group) AL: ad libitum, CR: caloric restriction, EX: exercise, HF: high-fat diet

Based on the target gene prediction and enrichment analyses, expression of representative predicted target mRNAs of some of the validated microRNAs were detected via RT-qPCR and these mRNAs are involved in all the treatments, including Elovl2, Lamp2, Atp6v0a1 and Wdr18 in CR, Wdr18 in EX and Atp6v0a1 and Wdr18 in HF (Fig. 7). The relationship between upstream microRNAs and the detected target mRNAs are listed in Table 2. The directions of the expression change detected by RT-qPCR were as expected.

**Table 2:** miRNA-target relationship of the detected mRNAs

Target gene	Upstream miRNAs	Predicted change
Elovl2	mmu-miR-802-5p, mmu-miR-96-5p	CR-Down
Lamp2	mmu-miR-802-5p, mmu-miR-96-5p	CR-Down
Atp6v0a1	mmu-let-7e-5p, mmu-miR-34a-5p	CR-Up, HF-Down
Wdr18	mmu-let-7e-5p, mmu-miR-34a-5p, mmu-miR-455-3p, mmu-miR-141-3p	CR-Up, EX-Up, HF-Down

## Discussion

It has been well known that lifestyle, such as caloric restriction, exercise and high-fat diet, has significant influence on health. Although there are many studies that have attempted to clarify the molecular processes, it has not been fully understand the underlying common or unique mechanisms. Therefore, identifying the underlying mechanism is crucial to determine new targets, personalize treatment methods and bring a promise of translatability to human health. In the present study, this is the first report that compares the microRNAs profile in livers from these three lifestyle modification mice models. In addition, we also predicted the potential functions of DE microRNAs by GO and KEGG analysis. With this knowledge, our findings provide us with an overall vision of microRNAs in the molecular impact of lifestyle on health as well as useful clues for future and thorough research of the role of microRNAs.

The different energy intake and consumption status of lifestyle modifications were presented in our prepared mice models as reported previously [19, 20]. Among lifestyle modifications, CR and endurance exercise can prevent or delay the onset of type 2 diabetes and metabolic syndrome, while high-fat diet induces obesity that leads to these diseases. The three lifestyle modification models guaranteed the miRNAs profiling results.

Many researchers commonly used microarrays to screen DE microRNAs in various pathophysiological processes [21]. Although an increasing number of studies applied next-generation sequencing (NGS) to perform comprehensive analyses of microRNA expression profiles, it has been demonstrated that NGS and microarray measurements give similar results [22]. In addition, in this study, 4 ~ 6 DE microRNAs identified by microarray in each lifestyle treatment were verified via RT-qPCR. These results confirmed the reliability of our data and provided a credible base for further study.

Some studies have shown that microRNAs are involved in the cellular and molecular mechanisms of lifestyle modifications [14–16]. However, most of the microRNA profiling studies of exercise focus on circulating microRNAs or microRNAs in skeletal muscle and heart [9, 14, 16]. Although there are some studies on microRNA profiling in liver of HF or CR [23–27], there is little comprehensive information regarding the similarities and differences of microRNAs profile in liver between these beneficial and detrimental lifestyles. Therefore, we examined the overall microRNAs expression in the liver of mice subjected to CR, EX and HF. In general, about half microRNAs were detectable in liver and the responses of microRNAs to these lifestyle modifications were relatively mild. On one hand, only a small portion were responded to lifestyle modifications; on the other hand, most of the DE microRNAs changed within a small range. Different from these results, in some diseases or physiological process, such as Parkinson’s Disease [28], fetal development [29], hepatocellular carcinoma [30], ischemia/reperfusion-induced acute kidney injury [31] and hepatitis C virus infection [32], there are more than one hundred DE microRNAs or the ranges of DE microRNAs change can be up to tens or more than one hundred folds. Among the three lifestyles, CR had the mildest impact on microRNAs, DE microRNAs in EX changed to the biggest range. Based on this result, to get the beneficial effects to health, maybe CR is a gentler choice. On the other side, most of the changes by the beneficial lifestyles were up-regulation, while the number of down- and up-regulated microRNAs by the detrimental lifestyle HF were about equal. More down-regulated microRNAs imply more up-regulated mRNA. It’s a possible way that HF disturbs homeostasis. In addition, our results showed some common DE microRNAs between different lifestyle modifications. For example, we found that miR-34a-5p was activated by HF and inhibited by CR. It has been reported that miR-34a was aberrantly elevated by HF and functionally involved into hepatic lipid metabolism [25, 33, 34]. In the brain of CR mice, there is a decreased expression of mmu-miR-34a [35]. Another example is mmu-miR-200b-5p, which was up-regulated by both CR and EX in the present study. Consistent with our findings, mmu-miR-200b-5p was also elevated in salivary post-running [36]. These results are fundamental and we undertook a more thorough and comprehensive analysis of potential microRNAs involved in the effects on health by lifestyle modifications.

To predict the potential functions of the DE microRNAs in present study, we performed GO and KEGG analyses on the predicted targets. The ontology comprises three distinct aspects of gene function: biological process (a biological objective to which the gene or gene product contributes), cellular component (the location in the cell where a gene product is active), and molecular function (the biochemical activity of a gene product at the molecular level) [17, 37]. KEGG is for understanding functional meanings of genes and genomes both at the molecular and higher levels [38, 39]. The GO and KEGG analysis showed that targets of the DE microRNAs in these lifestyle modifications were enriched in some common main functions, biochemical and signal transduction pathways, such as oxidation-reduction process and oxidoreductase activity, metabolic process and metabolic pathways, fatty acid metabolic process and fatty acid degradation, PPAR signaling pathway. These gene functions and pathways of the targets were also shown in some previous studies about lifestyle modifications. For example, exercise exerted profound changes in metabolism-associated genes, which encode proteins involved in oxidation, fatty acid transporter and fatty acid synthase [40]. PPARδ has also been observed in response to exercise [41]. The changes of the pathways, such as lipid metabolism, fatty acid degradation and metabolic pathways, have also been reported in CR [42, 43]. Studies have reported that HF has an important impact on the lipid metabolism process in rat liver [44]. The intake of a high-fat diet forces the body to maintain physiological balance by inhibiting fatty acid synthesis, promoting fatty acid oxidation, and accelerating fatty acid degradation. PPAR signaling pathway is also change significantly in rat liver after HF [45]. Besides, we also presented that targets of DE microRNAs in different lifestyle modification were also enriched in some different functions or pathways. For an instance, we found that CR altered expression of microRNAs implicated in regulating fatty acid oxidation, which is consistent with previous reports [42, 46]. Interestingly, we validated the expression change of a predicted target of mmu-miR-802-5p and mmu-miR-96-5p, Elovl2, in CR. Elovl2 is related with fatty acyl-CoA biosynthesis [47], while GO-MF term fatty-acyl-CoA binding was enriched in CR functions. Lamp2, another predicted target of mmu-miR-802-5p and mmu-miR-96-5p, belongs to autophagy–lysosome system and also reduces in liver of CR mice. A similar down-regulation of Lamp2 was reported by Junya Yamamoto and colleagues in mice livers after fasting [48].

Although there are some limitations in our study, such as a relatively small sample size, we confirmed some of the DE microRNAs and predicted targets with RT-qPCR. Differences of identified DE microRNAs do exist between our study and previous studies [23–27]. One possible reason is the differences of treatment, such as duration, age, diet ingredients; another possibility is difference between detection methods. Besides, although we observed inverse correlations between several microRNAs and their targets, direct evidences of repression by microRNAs on their targets need to be provided, and these mechanisms would have to be investigated further to gain more insight into these potential miRNA-target relationships. Cell culture experiments with overexpression or knockdown of microRNAs would enable us to elucidate this.

## Conclusions

In conclusion, this study provided for the first time a comprehensive and thorough comparison of microRNAs expression profiles in liver among beneficial and detrimental lifestyles, including CR, EX and HF. We presented similarity and differences of DE microRNAs among them. Besides, the data revealed that, through the interaction and regulation of related genes, DE microRNAs participate in related specific biological processes and pathways that may contribute to the effects of these lifestyles. While our findings provide us with an overall vision of microRNAs in the molecular impact of lifestyles on health, further studies are required to decipher the underlying molecular mechanisms of these DE microRNAs.

## Methods

### Ethics statement

This study was carried out in strict accordance with the recommendations in the Guide for the Care and Use of Laboratory Animals of the National Institutes of Health. The protocol was approved by the Biomedical Ethics Committee of Beijing Hospital and Beijing Institute of Geriatrics, Beijing, China. Staff veterinarians monitored mice on a regular basis, finding no pathogens. All efforts were made to minimize suffering.

### Animal models

Male C57BL/6 mice were purchased from the Vital River (Charles River China) at two months of age. After a one-week acclimation, all mice were randomly assigned to the following groups and treated for 3 months (*n* = 7 in each group): ad libitum, normal-fat diet group (AL); ad libitum, high-fat diet group (HF) [19, 20]; normal-fat diet and 30 ~ 35% caloric restriction as in AL group (CR) [46, 49, 50]; and ad libitum, normal-fat diet with exercise (EX). The mice were exercised on a motorized treadmill from EHSY (Shanghai, CN) for 30 min/day at 14 m/min, 5° incline, and 5 days/week for 3 months. Intensity of exercise is moderate and corresponds to 70 ~ 75% of maximal oxygen uptake [19, 51]. All the animals were housed at 21 °C in a 12-h light/12-h dark cycle (lights on at 7:00 am). We recorded the body weight and food intake once a week during the study. Body composition was measured by dual energy X-ray absorptiometry (DEXA, Discovery, HOLOGIC Com., MA, USA.). Anesthesia of the mice was performed with isoflurane before their euthanasia by cervical dislocation. The livers were removed, weighed, frozen in liquid nitrogen and stored at − 80 °C.

### RNA extraction

Livers were isolated, snap frozen in liquid nitrogen, ground into powder with mortar and pestle in liquid nitrogen and then stored at − 80 °C. Total RNA was isolated from 20 to 30 mg liver using TRIzol® reagent (Invitrogen Life Technologies, USA) and RNasey Mini Kit (Qiagen, Germany) following the manufacturer’s instructions. The residual DNA was removed by TURBO DNA free kit (Ambion Inc., UK). Yield and purity of RNA were determined by NanoDrop ND-1000 spectrophotometer (Nanodrop technologies, USA). RNA integrity and genomic DNA contamination were tested by denaturing agarose gel electrophoresis.

### Microarray

MicroRNA expression levels were assessed using a microRNA microarray (miRCURY LNA™ microRNA Array (v.11.0), Exiqon, Vedbæk, Denmark), based on the method of locked nucleic acid [52]. All chips were prepared according to the manufacturer’s instructions at KangChen Bio-tech (Shanghai, China). All probes with four calls were selected for assessing differential expression between groups. For each group, total RNAs from 3 mice were pooled with equal quantity to get one sample for microarray detection. The RNA samples were labeled using the miRCURY™ Hy3™/Hy5™ Power labeling kit and hybridized on the chip. Scanning was performed with the Axon GenePix 4000B microarray scanner. GenePix pro V6.0 was used to read the raw intensity of the image. The intensity of green signal on the chip was calculated after background subtraction and replicated spots on the same slide had been averaged by getting a median intensity. We used Median Normalization Method to obtain “Normalized Data”, Normalized Data = (Foreground-Background) /median, the median is 50% quantile of microRNA intensity which is larger than 50 in all samples after background correction. The low intensity differentially expressed (DE) microRNAs are filtered (which Foreground-Background intensities are all < 50 in two compared samples). The threshold value we used to screen Up and Down regulated microRNAs is Fold Change > = 1.5 compared to AL group.

### cDNA synthesis and real-time PCR

Validity of the microRNAs expression array was confirmed by RT-qPCR. A Mir-X microRNA First-Strand Synthesis Kit (Clontech Laboratories, Inc. CA, USA) was used to synthesize first-strand cDNA according to the manufacturer’s instructions. QPCRs of microRNAs were then conducted in iQ5 Real-Time PCR system (Bio-Rad Laboratories, Inc., CA, USA) using a Mir-X microRNA qRT-PCR SYBR Kit (Clontech Laboratories, Inc. CA, USA) (*n* = 5 in each group). The amplification program was as follows: 95 °C for 10s, 40 cycles at 95 °C for 5 s and 60 °C for 20s, with a final melting curve at 95 °C for 60s, 55 °C for 30s, and 95°Cfor 30s. A U6 snRNA, detected with primers supplied with the kit, was used as an internal control to calculate the relative expression of microRNAs using the 2^–ΔΔCt^ method.

Candidate predicted target mRNAs were also confirmed by RT-qPCR. First-strand cDNA was synthesized from total RNA using a 20 μl reverse transcription system (New England Biolabs, USA). QPCRs were conducted using a TB Green® Premix Ex Taq™ II Kit (Takara Bio Inc., Japan) (*n* = 5 in each group). The amplification program was as follows: 95 °C for 30s, 40 cycles at 95 °C for 5 s and 60 °C for 30s. After amplification, a thermal denaturing cycle was added as above. Hmbs was used as an internal control [50] to calculate the relative expression of target mRNAs using the 2^–ΔΔ^Ct method.

The primers were listed in Table 3 and were ordered from Life Technologies (Beijing, China) with their certificates of analysis.

**Table 3:** Primers for qRT-PCR validation of candidate miRNAs and mRNAs

	Gene	Primer
miRNA	mmu-miR-141-3p	CGCTAACACTGTCTGGTAAAGATGG
	mmu-miR-200b-5p	CATCTTACTGGGCAGCATTGGA
	mmu-miR-34a-5p	TGGCAGTGTCTTAGCTGGTTG
	mmu-miR-380-5p	CGATGGTTGACCATAGAACATGCG
	mmu-miR-409-3p	CCGAATGTTGCTCGGTGAACC
	mmu-miR-455-3p	GCAGTCCACGGGCATATACAC
	mmu-miR-487b-3p	CAATCGTACAGGGTCATCCACTT
	mmu-miR-683	CCTGCTGTAAGCTGTGTCCTC
	mmu-miR-802-5p	GGCCTCAGTAACAAAGATTCATCCTT
	mmu-miR-96-5p	GTTTGGCACTAGCACATTTTTGCT
	mmu-miR-99a-5p	CAACCCGTAGATCCGATCTTGTG
	mmu-let7e-5p	CGCTGAGGTAGGAGGTTGTATAGT
mRNA	Atp6v0a1	F: CCGAGGACGAAGTGTTTGACT R: ATCAGCAGGATAGCCACGGT
	Elovl2	F: CCTGCTCTCGATATGGCTGG R: AAGAAGTGTGATTGCGAGGTTAT
	Lamp2	F: TGTATTTGGCTAATGGCTCAGC R: TATGGGCACAAGGAAGTTGTC
	Wdr18	F: TGGTGTGGGAGCTTCATTCG R: CCCAGGCGCAGATGTAGTTC
	Hmbs	F: ATGAGGGTGATTCGAGTGGG R: TTGTCTCCCGTGGTGGACATA

### Target predictions for differentially expressed microRNAs

Target prediction for the DE microRNAs was performed using Targetscan Mouse release 7.1, miRanda, miRDB and miRWalk2.0 [53–56]. To improve the accuracy of target gene prediction and reduce the rate of false positives, the intersections of the output results of at least three algorithms were used as prediction results for the DE microRNAs. We filtered the vast list of potential targets by employing the PaGenBase database to identify tissue-specific gene (mRNA) targets [57].

### Target gene annotation, enrichment and pathway analysis

The predicted targets of DE microRNAs of each group were separately submitted to DAVID for annotation and enrichment analyses. The main components of annotation in Gene Ontology (GO) mainly provided the cellular locations and biological functions of validated microRNA targets [17]. The GO-biological processes, GO-molecular function and GO-cellular component analyses were performed using Fisher’s exact test and the χ^2^ test, where both the Expression Analysis Systematic Explorer and the False Discovery Ratio (FDR) were calculated to correct the *p* value. Only terms with both a p value and an FDR < 0.05 were considered to be significant. Pathway analysis was based on the Kyoto Encyclopedia of Genes and Genomes (KEGG) database [18]; similarly, we used Fisher’s exact test and the χ^2^ test to identify significant pathways, and terms with both a p value and an FDR < 0.05 were considered to be significant.

### Statistical analysis

Data are expressed as group mean ± standard error (SE). SPSS software (version 17.0) was used for statistical analysis. Means of two groups were compared and analyzed using the Student’s t-test. One-way analysis of variance (ANOVA) was used to estimate difference among groups, followed by Tukey’s post-hoc test. Differences were reported as statistically significant when *p* < 0.05. GraphPad Prism 6 (GraphPad Software, USA) was used for graph plotting.

## Supplementary Information

**Additional file 1.**

## Data Availability

The datasets generated and/or analysed during the current study are available in the GEO repository with accession numbers GSE148148 (https://www.ncbi.nlm.nih.gov/geo/query/acc.cgi?acc=GSE148148).

## Abbreviations

AL: Ad libitum
ANOVA: Analysis of variance
BP: Biological process
CC: Cellular component
CR: Caloric restriction
DE: Differentially expressed
EX: Exercise
FDR: False Discovery Ratio
GO: Gene Ontology
HF: High-fat diet
KEGG: Kyoto Encyclopedia of Genes and Genomes
MF: Molecular function
NGS: Next-generation sequencing
RT-qPCR: Reverse transcription quantitative real-time polymerase chain reaction
